# Correlation Between Stone Composition and Serum Metabolic Abnormalities in Recurrent Renal Calculi

**DOI:** 10.7759/cureus.96535

**Published:** 2025-11-10

**Authors:** Numan Alam, Riaz Ali Khan, Suffia Hayee, Aziz Ul Wahab, Asif Iqbal

**Affiliations:** 1 Urology, District Headquarter Hospital (DHQ), Mardan, PAK; 2 Urology, District Headquarter Hospital (DHQ), Daggar, PAK; 3 General Surgery, Combined Military Hospital Rawalpindi, Rawalpindi, PAK; 4 Urology, District Headquarter Hospital (DHQ), Timergara, PAK; 5 Nephrology, District Headquarter Hospital (DHQ), Daggar, PAK

**Keywords:** metabolic abnormalities, recurrent kidney stones, renal calculi, serum biochemistry, stone composition, urolithiasis

## Abstract

Background: Renal stone disease is one of the most prevalent urological conditions, often resulting from a complex interplay of metabolic, dietary, and environmental factors. Despite its multifactorial origins, recurrent renal calculi continue to pose a significant burden in urology. Identifying underlying biochemical abnormalities is essential to developing preventive strategies aimed at reducing the risk of recurrence.

Objective: To assess the discrepancy between renal calculi composition and serum biochemical anomalies to determine the metabolic abnormalities indicative of recurrent renal calculi.

Methods: This prospective observational study was conducted at District Headquarter Hospital, Mardan, Pakistan, over a two-year period (February 2022 to January 2024) on 120 consecutive adult patients with recurrent renal or ureteric stones. The study included analyses of serum calcium, phosphate, uric acid, vitamin D, parathyroid hormone (PTH), and bicarbonate levels. Stones were analyzed using Fourier-transform infrared spectrography after being surgically removed or voided. The clinical correlation between the biochemical serum anomaly, type of calculi, and recurrence was evaluated using the chi-square test and logistic regression models.

Results: The most common composition of stones was calcium oxalate, constituting 53.3% of the total number of stones, followed by mixed calcium oxalate-phosphate and uric acid stones, constituting 23.3% and 13.3%, respectively. Metabolically, the most common abnormalities were vitamin D deficiency (55.8% of participants), followed by hyperuricemia (18.3%) and hypercalcemia (11.7%). Uric acid stones were significantly influenced by serum uric acid levels (p < 0.001), and vitamin D deficiency was associated with mixed calcium oxalate-phosphate stones (p = 0.032).

Conclusion: The presence of particular serum metabolic abnormalities is significantly associated with specific stone compositions as well as with particular patterns of recurrence. Addressing recurrent renal stone disease sufficiently should include a thorough metabolic evaluation and correction of vitamin D deficiency and hyperuricemia, along with other modifiable metabolic derangements, to minimize the occurrence of future stones.

## Introduction

Recurrent urinary stone disease is a global health issue, particularly in urology, and is also very expensive, especially the calcium-oxalate type stones. Epidemiologic and stone analysis studies suggest that the primary predominant stone type in most geographic and population ranges is calcium-oxalate stones, but the composition is mixed [1].

The formation of stones is greatly influenced by identifiable abnormal metabolic parameters. The most common abnormal metabolic parameters in stone formers include low urine volume, hypercalciuria, hypocitraturia, hyperoxaluria, and hyperuricosuria. These parameters are all targetable with dietary and pharmacologic intervention in an attempt to reduce the risk of recurrence [2].

Some metabolic parameters are classically (and improperly) associated with certain stone types, such as hyperuricosuria and low urine pH with uric acid stones, infection with struvite, and hypercalciuria with calcium phosphate/oxalate stones. Correlating stone composition with metabolic parameters in urine and serum will elucidate the pathogenic mechanisms in that individual, allow for a tailored preventive strategy, and provide the opportunity to predict patterns of recurrence [3]. However, more contemporary studies emphasize the prevalence of mixed stones and the need for population-specific data to refine the predictive associations [4,5].

Therefore, guideline panels, along with recent narrative reviews, have advocated for metabolic evaluation, which includes serum chemistries along with two 24-hour urine collections or equivalent evaluations with repeat assessments in the context of recurrent stones or recurrent stone formation due to the significant effect of evidence-based intervention on recurrence [6]. Even with these recommendations, the relationship between certain serum metabolic anomalies and the composition of stones, particularly in recurrent stone cohorts, remains poorly defined in various populations [7].

Emerging metabolomics and observational studies have started to delineate systemic metabolic signatures related to stone disease and have exposed associations between certain stone phenotypes and cardiometabolic conditions, such as obesity, hyperuricemia, and insulin resistance, indicating potential benefits of serum metabolic profiling in complementing the conventional urine-based assessments [8,9]. However, the absence of practical, clinically oriented correlation studies in the literature remains, which involves the prediction of stone composition and recurrence risk in real-world single-center cohorts based on clinically accessible serum and urine tests.

Thus, the current study focuses on the relationship between the serum metabolic abnormalities and stone composition (via standard stone analysis) in recurrent renal calculi patients at our institution. We expect specific serum biochemical discrepancies to align with predominant stone types and patterns of recurrence, eventually helping us to shape tailored prevention and monitoring procedures. Moreover, there is a notable gap in local data on this subject, further strengthening the rationale for conducting this study to provide region-specific insights into metabolic risk factors and stone composition patterns.

## Materials and methods

This two-year prospective observational study was carried out at the District Headquarter Teaching Hospital, Mardan, Pakistan, from February 2022 to January 2024. Adult cases between the ages of 18 and 70 presenting with recurrent renal or ureteric calculi, which were diagnosed via ultrasound or non-contrast computed tomography (NCCT), were recruited for the study. The study’s sample size was estimated from the previously determined prevalence of 70-80% metabolic abnormalities from recurrent renal stone formers [10]. We estimated the prevalence to be 75% with a 95% confidence level and 8% margin of error, which gives the sample size of 110. Considering the incomplete metabolic assessment and data attrition, 120 patients were taken for the study. All the patients who fulfilled eligibility requirements and gave informed consent during the study period were enrolled in a consecutive manner till this number was achieved. The recurrent stone disease was defined as two or more documented episodes of stone formation occurring more than six months apart. Patients with solitary kidneys or lower than stage 3 chronic kidney disease, urinary tract infection, primary hyperparathyroidism, gout, or treatment with calcium or uric acid-modifying drugs, thiazides, allopurinol, or corticosteroids were excluded.

Clinical histories were taken in detail, including history of dietary habits, fluid intake, family history of urolithiasis, and other co-morbid conditions (diabetes, hypertension, and obesity). Anthropometric data in regard to weight, height, and body mass index (BMI) were also collected and recorded. Blood samples were obtained after an overnight fast to determine calcium, phosphorus, uric acid, creatinine, sodium, potassium, bicarbonate, and parathyroid hormone (PTH) levels using standard automated analyzers. Vitamin D (25-hydroxycholecalciferol) levels were determined by chemiluminescent immunoassay. Urine samples were analyzed for pH, specific gravity, and microscopic crystals, and 24-hour urine samples were collected (when possible) for quantitative calcium, uric acid, oxalate, citrate, and phosphate excretion.

Stone retrieval occurred through spontaneous passage, ureteroscopy, percutaneous nephrolithotomy, and extracorporeal shock wave lithotripsy. Completed stone retrieval involved washing, drying, and composition analysis using Fourier-transform infrared spectroscopy (FTIR), which detects major and minor stone constituents. Stones are classified based on the results of the FTIR analysis and are described as calcium oxalate, calcium phosphate, uric acid, struvite, cystine, or mixed type.

The data collection and compilation process utilized a prestructured spreadsheet. Associations of serum biochemical and urine findings with various stone compositions were evaluated. Continuous data were summarized as mean ± standard deviation (SD) and categorical data as frequencies and percentages. Mean comparisons of biochemical parameters and reference values were performed using a one-sample t-test for continuous variables. Associations of categorical variables, including stone composition and metabolic abnormalities, were assessed using the chi-square test. Binary logistic regression was used to evaluate the predictors of stone recurrence. Cases with incomplete biochemical or stone composition data were excluded from specific analyses involving those variables. However, such cases were retained for analyses where complete data were available, following a pairwise deletion approach to preserve statistical power. A p-value of ≤0.05 was considered statistically significant. All analyses were performed using IBM SPSS Statistics for Windows, Version 26 (Released 2019; IBM Corp., Armonk, New York, United States).

Ethical clearance for the study was obtained from the Ethics Review Board (reference # 471/MS/Supdtt, dated 22-1-2022). All study participants provided informed consent in writing prior to inclusion in the study. The study adhered to the principles of the Declaration of Helsinki.

All laboratory investigations were performed using standard, validated clinical chemistry methods that are freely available for research and diagnostic use. No copyrighted scales, scoring systems, or proprietary instruments requiring special licenses were employed. Stone analysis was conducted using FTIR, a universally accepted, non-proprietary technique.

## Results

The average age was 42.6 ± 11.8 years (19-68 years), and the male-to-female ratio was 2.3:1. The average BMI was 26.4 ± 3.8 kg/m^2^, and 38.3% were classified as overweight or obese. A family history of urolithiasis was documented in 29.1% of the study population, while 32.5% reported inadequate daily fluid intake (<2 L/day) (Table 1).

**Table 1:** Baseline demographic and clinical characteristics of the study population

Parameter	Value
Mean age (years)	42.6 ± 11.8
Male: Female	83: 37
Mean BMI (kg/m^2^)	26.4 ± 3.8
Overweight/obese (%)	38.3
Family history of urolithiasis (%)	29.1
Inadequate fluid intake (%)	32.5
Diabetes mellitus (%)	21.6
Hypertension (%)	18.3
Mean stone episodes per patient	2.8 ± 0.9

Among the different types of stones retrieved, the most common were calcium oxalate stones (64, 53.3%). This was followed by mixed calcium oxalate-phosphate stones found in 28 (23.3%) patients, uric acid stones in 16 (13.3%) patients, and struvite stones in eight (6.7%) patients. Cystine stones were the rarest and were found in four (3.3%) patients (Table 2).

**Table 2:** Distribution of stone composition among study participants

Stone Type	n (%)
Calcium oxalate	64 (53.3)
Mixed calcium oxalate-phosphate	28 (23.3)
Uric acid	16 (13.3)
Struvite	8 (6.7)
Cystine	4 (3.3)

Serum biochemical analysis indicated that hypercalcemia was present in 14 (11.7%) patients, hyperuricemia in 22 (18.3%) patients, and hypovitaminosis D in 67 (55.8%) patients. The average serum calcium level was 9.4 ± 0.6 mg/dL, uric acid was 6.7 ± 1.5 mg/dL, and vitamin D was 22.1 ± 8.7 ng/mL. Elevated PTH levels were found in 10 (8.3%) patients, and 12 (10%) patients had mild metabolic acidosis (serum bicarbonate <22 mmol/L) (Table 3).

**Table 3:** Distribution of serum metabolic abnormalities Mean biochemical parameters compared with reference values using a one-sample t-test. Data are expressed as mean ± SD; p ≤ 0.05 is considered statistically significant. PTH: parathyroid hormone

Parameter	Mean ± SD	Abnormal (%)	Statistical Test	Test Statistic (t/χ^2^)	P-value
Serum calcium (mg/dL)	9.4 ± 0.6	11.7	One-sample t-test	t = 2.41	0.018
Serum uric acid (mg/dL)	6.7 ± 1.5	18.3	One-sample t-test	t = 3.26	0.001
Serum phosphate (mg/dL)	3.4 ± 0.5	7.5	One-sample t-test	t = 1.63	0.106
Vitamin D (ng/mL)	22.1 ± 8.7	55.8	One-sample t-test	t = -5.92	<0.001
PTH (pg/mL)	54.7 ± 18.6	8.3	One-sample t-test	t = 1.09	0.278
Serum bicarbonate (mmol/L)	23.8 ± 2.1	10.0	One-sample t-test	t = -2.15	0.034

A statistical association was reported between the levels of serum uric acid and the formation of uric acid stones (p < 0.001). Likewise, hypercalcemia was directly associated with the development of calcium-containing stones (p = 0.008). Vitamin D-deficient patients had mixed calcium oxalate-phosphate stones more frequently than patients with normal vitamin D levels (p = 0.032). No significant relationship was found between PTH elevation and any particular stone subtype (p = 0.176) (Table 4).

**Table 4:** Correlation between stone composition and serum metabolic abnormalities The association between stone type and serum metabolic abnormalities was analyzed using the chi-square test. P ≤ 0.05 is considered statistically significant. NS: not significant; PTH: parathyroid hormone

Serum Parameter	Stone Type Most Associated	Statistical Test	χ^2^ Value	P-value	Significance
Serum calcium ↑	Calcium oxalate	Chi-square	7.02	0.008	Significant
Serum uric acid ↑	Uric acid	Chi-square	15.64	<0.001	Highly significant
Vitamin D ↓	Mixed calcium oxalate–phosphate	Chi-square	4.61	0.032	Significant
PTH ↑	None specific	Chi-square	1.83	0.176	NS
Serum bicarbonate ↓	Uric acid	Chi-square	3.88	0.049	Significant

In the course of the mean follow-up period of 18.4 ± 4.6 months, 34 (28.3%) of the patients had at least one recurrence. Having two or more metabolic abnormalities was associated with a higher rate of recurrence (38.2%) when compared with patients with a single or no abnormality (17.9%) (p = 0.015). Logistic regression yielded hyperuricemia (odds ratio (OR) 3.4; 95% CI 1.4-8.2; p = 0.007) and vitamin D deficiency (OR 2.1; 95% CI 1.0-4.3; p = 0.046) as the greatest predictors of recurrence of hyperuricemia (Table 5).

**Table 5:** Association of metabolic abnormalities with stone recurrence The association between metabolic abnormalities and stone recurrence was analyzed using the chi-square test and binary logistic regression (Wald χ^2^ test). CI: confidence interval; p ≤ 0.05 considered statistically significant.

Variable	Recurrence (%)	Statistical Test	Test Statistic	Odds Ratio (95% CI)	P-value
Hypercalcemia	35.7	Logistic regression (Wald test)	χ^2^ = 1.66	1.9 (0.7-5.2)	0.198
Hyperuricemia	45.5	Logistic regression (Wald test)	χ^2^ = 7.31	3.4 (1.4-8.2)	0.007
Vitamin D deficiency	33.8	Logistic regression (Wald test)	χ^2^ = 3.99	2.1 (1.0-4.3)	0.046
≥2 abnormalities	38.2	Chi-square	χ^2^ = 5.89	2.8 (1.2-6.7)	0.015

## Discussion

The current study assessed stone composition and serum metabolic abnormalities in patients with recurrent renal calculi in our setting. As noted in the literature, our findings indicated that calcium oxalate continues to be the most prominent type of stone. Recent meta-analyses state that calcium oxalate stones constitute 60% to 80% of all urinary stones, followed by the mixed calcium phosphate and uric acid stones [10,11]. In our study, calcium oxalate stones constituted 53.3% of all cases, slightly less than the upper global range. These differences from the upper global range are likely due to differences in diet, water minerals, and the genetics of calcium and oxalate stones forming/calculating in urine. In addition, the relatively high number of mixed calcium oxalate-phosphate stones (23.3%) indicates that this population likely has overlapping metabolic disturbances, including vitamin D deficiency and mild hypercalcaemia, which, in turn, likely facilitates phosphate to oxalate stones. The male predominance in our cohort is consistent with the well-established differences in the epidemiology of stone disease that are attributed to men’s higher urinary calcium and oxalate excretion as well as differences in diet and lifestyle [12].

Vitamin D deficiency, hyperuricemia, and hypercalcemia were noted as the most frequent biochemical derangements in the recurrent stone formers in our study. These findings are consistent with the reports of Girón-Prieto et al. [13], which emphasized the multifactorial nature of systemic metabolic derangements in the pathogenesis of nephrolithiasis. Although hypercalciuria is routinely described as the most prevalent metabolic risk factor, the role of serum calcium and vitamin D in the formation of stones has not been clearly delineated. Several investigators have described how low vitamin D levels, through the stimulation of PTH secretion, may lead to bone resorption and urinary calcium loss, thus creating a greater risk for calcium oxalate-phosphate nephrolithiasis [14,15].

The relationship between serum uric acid levels and uric acid stone formation, as reflected in our findings, has also been reported by Deligiannis et al. [16]. This consolidates the connection between hyperuricemia, low urine pH, and uric acid stone formation. High levels of uric acid also promote the urinary supersaturation of calcium oxalate, as it serves as a crystallization nidus, which explains the association with mixed-type stones in some patients. This suggests the presence of a calcium and uric acid stone combined metabolic pathway, as noted by Ferraro et al. [17].

In our study, the overall recurrence rate was 28.3% with a mean follow-up period of 18 months. This figure is similar to other published studies with recurrence rates within two years of 20-35% [18,19]. Recurrence rates also vary by subgroup. A higher recurrence rate of 38.2% was observed in patients with two or more derangements, as opposed to 17.9% in those with one or none (p = 0.015). The presence of multiple metabolic disturbances cumulatively contributes to stone recurrence and emphasises the need for thorough metabolic assessment and correction. The recurrence potential attributed to hyperuricaemia and vitamin D deficiency should be noted and can be targeted using dietary and pharmacological approaches. These findings have important clinical implications, as they emphasize the need for routine metabolic evaluation and early correction of modifiable factors such as hyperuricemia and vitamin D deficiency to reduce recurrence risk. However, potential confounding factors, such as variability in dietary intake, hydration status, and seasonal vitamin D fluctuations, may have influenced biochemical results and should be considered when interpreting these associations.

Metabolic screening remains valuable for assessing recurrent stone formers, as set out in the American Urological Association (AUA) Guidelines [20]. Identification of metabolic abnormalities, such as dietary sodium restriction, hydration, correction of vitamin D deficiency, and uric acid lowering, allows for the focused management of these abnormalities and significantly lowers the risk of recurrence.

The strength of this study lies in its prospective design and the use of FTIR for detailed analysis of stone composition. However, several limitations should be acknowledged. This was a single-center study with a relatively small sample size, which may limit the generalizability of the findings. Metabolic urine data were available only for a subset of participants, and while the follow-up duration was adequate for evaluating short-term recurrence, it may be insufficient to capture late recurrences. Future multi-center studies incorporating both serum and 24-hour urinary metabolic analyses are warranted to provide deeper mechanistic insights and develop individualized preventive strategies for recurrent stone formers.

Metabolic evaluations should become a routine practice integrated into the diagnostic and preventative strategies for all recurrent and high-risk stone formers. Individualized therapeutic approaches should be implemented based on the results of serum and, when possible, 24-hour urinary biochemistry evaluations. Implementing corrective measures that include adequate hydration, dietary adjustments that include the reduction of salt and animal protein, and the maintenance of adequate vitamin D levels, along with pharmacological treatment for hyperuricemia or hypercalciuria, should, without hesitation, be considered. Longitudinal studies with repeated biochemical assessments can provide valuable information to ascertain treatment adherence and its results. Future multicentric studies that unite metabolic, dietary, and genomic information will likely enhance our understanding of stone formation and aid the construction of tailored, predictive models aimed at preventing stone recurrence.

## Conclusions

This study establishes significant associations between specific serum metabolic abnormalities and recurrent renal stone composition, identifying vitamin D deficiency and hyperuricemia as key modifiable predictors of recurrence. Targeted metabolic correction may therefore play a pivotal role in preventing future stone formation.
